# 375. The Effect of Inhaled Interferon Beta-1a (SNG001) Treatment Compared to Placebo on Lung Antiviral Biomarkers and Viral Clearance in Chronic Obstructive Pulmonary Disease (COPD) Patients With Respiratory Virus Infections

**DOI:** 10.1093/ofid/ofad500.445

**Published:** 2023-11-27

**Authors:** Phillip D Monk, Jody L Brookes, Victoria J Tear, Toby Batten, Clare Newall, Marcin Mankowski, Michael G Crooks, Dave Singh, Rekha Chaudhuri, Kerry Lunn, Sophie Reynolds, Sarah Dudley, Felicity Gabbay, Stephen T Holgate, Ratko Djukanovic, Tom Wilkinson

**Affiliations:** Synairgen Research Ltd, Southampton, England, United Kingdom; Synairgen Research Ltd, Southampton, England, United Kingdom; Synairgen Research Limited, Southampton, England, United Kingdom; Veramed Ltd, Twickenham, England, United Kingdom; Veramed Ltd, Twickenham, England, United Kingdom; tranScrip, Wokingham, England, United Kingdom; Hull York Medical School, University of Hull, Hull, England, United Kingdom; University of Manchester, Manchester, England, United Kingdom; University of Glasgow, Glasgow, Scotland, United Kingdom; Synairgen, southampton, England, United Kingdom; Synairgen Research Ltd, Southampton, England, United Kingdom; Synairgen Research Limited, Southampton, England, United Kingdom; Transcrip, Winnersh, England, United Kingdom; University of Southampton, Southampton, England, United Kingdom; Faculty of Medicine, University of Southampton, SOUTHAMPTON, England, United Kingdom; University of Southampton, Southampton, England, United Kingdom

## Abstract

**Background:**

Respiratory viral infections (RVIs) are major drivers of chronic obstructive pulmonary disease (COPD) exacerbations. Interferon beta (IFN-β) is key in host defence against viruses but can be suppressed by virus or host factors locally at the site of infection. Inhalation of SNG001 (IFN-β-1a nebuliser solution) aims to restore lung IFN-β levels. SG015 (NCT03570359) was a randomized, placebo-controlled Phase 2 clinical study of inhaled SNG001 conducted in COPD patients. Here we describe lung antiviral biomarker and sputum viral clearance data from Part 2 of the study which was conducted in patients with a confirmed RVI.

**Methods:**

109 COPD patients with worsening symptoms and a positive respiratory viral test were randomized 1:1 to SNG001 or placebo once-daily for 14 days in two Groups: A (no moderate exacerbation); B (moderate COPD exacerbation [i.e.,acute worsening of respiratory symptoms treated with antibiotics and/or oral corticosteroids]). Sputum samples were collected on days 1, 4, 7, 10, 13, 17 and 28 for analysis of lung antiviral biomarker responses (interferon-stimulated genes (ISGs): Mx1, OAS1 and CXCL10) and lung viral load by RT-qPCR.

**Results:**

Mx1 and OAS1 sputum cell gene expression were significantly upregulated on day 7, 10 and 13 (p< 0.05) overall and in Groups A and B with SNG001 treatment compared to placebo. CXCL10 sputum cell gene expression was significantly upregulated in the overall population with SNG001 treatment compared to placebo on days 7 and 10, in Group B on days 7, 10 and 13, and there was no significant difference in Group A. Patients had a broad range of RVIs, the most common being human rhinovirus. A *post-hoc* analysis was therefore conducted in the subgroup of patients who had detectable rhinovirus viral load in sputum. By Day 4 the proportion of patients receiving SNG001 who had detectable rhinovirus reduced to 40.0% (compared to 94.7% of patients receiving placebo; p=0.052), with a further reduction to 20.0% on Day 7 (versus 89.5% receiving placebo; p=0.014).

**Conclusion:**

Inhaled SNG001 upregulated lung antiviral defenses as assessed using sputum cell biomarker responses and accelerated viral clearance, supporting the proposed mechanism of action as an antiviral treatment for severe viral lung infections.

**Disclosures:**

**Phillip D. Monk, PhD**, Synairgen Research Plc: Employee of Synairgen Research Plc and has options on shares|Synairgen Research Plc: Stocks/Bonds **Jody L. Brookes, BSc**, Synairgen Research Ltd: Share options **Victoria J. Tear, PhD**, Synairgen Research Ltd.: Stocks/Bonds **Marcin Mankowski, MD MFPM (Dis)**, Multiple companies: Advisor/Consultant|Synairgen: Advisor/Consultant **Michael G. Crooks, MBChB (hons), MD, FRCP**, AstraZeneca: Advisor/Consultant|AstraZeneca: Grant/Research Support|AstraZeneca: Honoraria|Chiesi: Advisor/Consultant|Chiesi: Honoraria|Gilead: Honoraria|Synairgen: Advisor/Consultant **Dave Singh, MD**, AstraZeneca: Advisor/Consultant|Chiesi: Advisor/Consultant|gsk: Advisor/Consultant|Novartis: Advisor/Consultant|Orion: Advisor/Consultant|Pulmatrix: Advisor/Consultant|Sanofi: Advisor/Consultant|Synairgen: Advisor/Consultant|Synairgen: Grant/Research Support|Therevance: Advisor/Consultant **Rekha Chaudhuri, MD**, AstraZeneca: Grant/Research Support|AstraZeneca: Honoraria|Chiesi: Honoraria|GSK: Honoraria|Novartis: Honoraria|Sanofi: Honoraria|Teva: Honoraria **Sarah Dudley, N/A, PhD**, Synairgen Plc: Employed by Synairgen Research Ltd which is a subsidiary of Synairgen Plc|Synairgen Plc: Stocks/Bonds **Felicity Gabbay, MbChb**, Synairgen: Board Member **Stephen T. Holgate, FMedSci, MD**, Synairgen Research Plc: Board Member|Synairgen Research Plc: Stocks/Bonds **Ratko Djukanovic, MD**, GlaxoSmithKline: Advisor/Consultant|GlaxoSmithKline: Honoraria|KyMab: Advisor/Consultant|Sanofi: Advisor/Consultant|Synairgen: Advisor/Consultant|Synairgen: Stocks/Bonds **Tom Wilkinson, PhD, PhD**, Synairgen: Advisor/Consultant|Synairgen: Grant/Research Support|Synairgen: Honoraria

